# Cancrum Oris

**Published:** 1852-10

**Authors:** 


### ARTICLE XV.

### *Cancrum Oris.*

Pathologists have not, as yet, determined how it comes to pass, that mercury has a direct action on the salivary glands, and produces more or less severe stomatitis; nor is it much clearer why, under certain circumstances, the mouth of young subjects is attacked with a gangrenous inflammation which often destroys the patient. Whatever may be the final cause of the latter phenomenon, it is of a very important nature, both to the physician and the surgeon; for in this affection, as well as in a few others, medicine and surgery are closely blended.

We are accustomed to look for cancrum oris in children aged from five to ten years, or from two to eight years, but it would appear from the age of the patient (thirteen years) lately under the care of Dr. Burrows, that a very early age is not an invariable circumstance. Adults, however, seem quite free from this peculiar affection, for it is stated by Taupin, that in a total number of 1900 cases attended by him, at the Hotel Dieu at Paris, in 1837, only one adult suffered with cancrum oris. The latter manifestation is generally said to attack children exposed for some length of time to debilitating causes; and what Richter calls the metastatic species, supervenes on acute exanthematous diseases; it is likewise maintained that measles take the lead as a predisposing cause. To these opinions should be added, as will become apparent by the subjoined case, that common continued fever may be accompanied by gangrene of the mouth, though it would seem, that in such cases, the ulcerative process does not go so far as to cause the perforation of the cheek.

That the disease is very dangerous, and frequently proves fatal, has been acknowledged by all observers, the proportion of deaths depending very probably on the variety of the affection, namely, whether it be: 1st, peculiar to infants at the breast; 2nd, attacking children enfeebled by some previous disease; or, 3rd, confined to the cheeks and lips (Cumin.) We now beg a few moments attention to the following case.

Emma H——, thirteen years of age, was admittted into Faith ward, under the care of Dr. Burrows, Dec. 18, 1850. Her countenance is pale, languid, and heavy, the pupils dilated, the conjunctivæ suffused; the skin is warm and rather moist, but no rash or sudamina can be observed upon it. The tongue presents a dry and brown fur in its centre, the abdomen is soft, moderately painful on pressure in the lower portion, and especially round the umbilicus.

Alvine evacuations have been liquid, dark and bilious, there has been some vomiting, and the patient states that she has been ill three weeks, the complaint having begun with pain in the limbs and back; delirium had set in two days before admission, and purging and vomiting had continued for the same period.

10*

Dr. Burrows ordered wine and beef-tea; the hair to be shaved, and cold lotion applied to it, if necessary; four leeches to abdomen, and a mercurial purgative. From this period, to the twelfth day after admission, the patient went through the different phases of mild typhoid fever without maculæ, when she appeared much improved; but the right cheek was now noticed to be swollen, the patient having the night previously been rather restless. The patient complained, however, of no pain, though the face continued to swell, and was becoming red. She was ordered to take chlorate of potash with bark, and to use a gargle of chloride of soda. Mr. Stanley being consulted, advised the application of nitric acid, if the cheek did not improve.

There was now noticed, on the internal aspect of the cheek, a grayish slough, which posteriorly looked quite black. The pulse was 136, with tolerable volume; and the bowels relaxed. Wine, beef-tea, &c., were ordered; and Mr. Lloyd having likewise seen the patient, suggested applications of nitrate of silver to the gangrenous part. On the eighth day after the appearance of the gangrenous stomatitis, the cheek was reduced in size, and less red; in the centre a circle, about the diameter of a shilling, was noticed; it was soft to the touch, the parts around being firm and indurated. The slough within the mouth was of a gray color, and the edges of the sore connected with it very slightly inflamed. Dr. Burrows now prescribed ammonia and bark, and a poultice to the face. On the tenth day, an offensive slough of considerable size was detached from *the mouth*; the cheek became much diminished in bulk, and presented a circumscribed patch of dusky redness, over which the skin appeared about to desquamate. The patient took her food in the meanwhile, though with reluctance, as *swallowing* gave her much pain. The cheek gave the feeling of great tenuity at the affected spot; within the mouth was seen a deep and excavated ulcer, occupying the situation of the *two last upper and lower molar teeth*, with a small portion of slough remaining anteriorly.

On the twelfth day, the left commissure of the lips became much fissured. The patient now used, within the mouth, a lotion of fifteen grains of chlorate of potash in decoction of bark, and the wine and nourishing diet were continued. The interior of the ulcer was covered with a gray slough, and of the thickness of the cheek at that point nothing but the integument seemed to be left, the *second bicuspid tooth having dropped out.* On the sixteenth day another tooth from the upper jaw fell out, and on the twentieth pain was complained of; the gangrenous spot was darker externally, and the cuticle dry and desquamating over it. The patient, however, opened her mouth with less difficulty, and the sores on the left angle of the mouth were healing.

On the twenty-third day, after the accession of the gangrene, the dark spot on the face was situated at the base of a considerable depression on the side of the cheek; and around the darkest portion a circular superficial crack had made its appearance, enclosing a space about a quarter of an inch in diameter; and the part so enclosed resembled a scab. From this time an improvement began to take place; the size of the sore within the mouth diminished, and was covered with a white slough, resembling boiled albumen. Externally, the superficial crack was a little deeper, and somewhat tender to the touch.

On the thirtieth day the patient's appearance was much improved. On the right cheek a depression remained, slightly dusky in appearance, tender to the touch, but not feeling so thin as formerly. Another of the upper teeth now dropped out. On the thirty-third day the cheek, externally, presented a smaller depression, as well as a less dusky appearance; and internally, the ulcer was gradually contracting. On the fortieth day the ulcerated surface within the mouth had regained its mucous lining, and appeared healthy; though on its margins, and in the alveolar depressions whence the *molar teeth* had fallen out, there was yet a whitish gray foul slough. Still the above mentioned lotion has been used up to the present time. A complication of some importance, which occurred about this period, consisted of pain, redness, and swelling of the elbow of the right arm, as well as a certain amount of deafness; these symptoms were, however, removed by the usual means.

On the forty-third day there still remained a small slough on the posterior portion of the original gangrenous sore, and the alveolar portion of the upper jaw (the three molar teeth of which were absent) became exposed, and the bone necrosed. On the fiftieth day the patient sat up, slept well, and enjoyed a good appetite. No trace of ulceration remained within the mouth; the alveoli, however, were still in a necrosed state.

An evident hysterical re-action now took place, a circumstance easy of explanation at the patient's age, and she would frequently burst into tears. She was discharged in a favorable condition, but the change of scene and objects seemed, on her return home, to act powerfully on her debilitated frame; for, when with her friends, she became delirious, and was much excited. These symptoms continued for several days, and were treated by purgatives, low diet, &c. The former took full effect; large quantities of scybala were passed, and the patient rapidly regained full consciousness and tolerable health. A large portion of the superior maxillary bone came away about nine days after her discharge, and another loose piece will have to be removed before the mouth can be pronounced well.

The favorable turn which this case has taken, will show that deterging and caustic applications, combined with tonic medicine and stimulants, will conquer the fatal tendency of this dangerous affection. It will be perceived how close at hand was the actual perforation of the cheek; the phagedenic process, however, was luckily arrested, but the osseous texture did not escape; and the necrosis of the alveoli will bear a lasting testimony to the severity of the morbid phenomena.—*Lancet.*

